# In Vitro Cytotoxicity of Oleanolic/Ursolic Acids-Loaded in PLGA Nanoparticles in Different Cell Lines

**DOI:** 10.3390/pharmaceutics11080362

**Published:** 2019-07-24

**Authors:** Amélia M. Silva, Helen L. Alvarado, Guadalupe Abrego, Carlos Martins-Gomes, Maria L. Garduño-Ramirez, María L. García, Ana C. Calpena, Eliana B. Souto

**Affiliations:** 1 Centre for Research and Technology of Agro-Environmental and Biological Sciences (CITAB), University of Trás-os Montes e Alto Douro (UTAD), Quinta de Prados, 5001-801 Vila Real, Portugal; 2 Department of Biology and Environment, UTAD, Quinta de Prados, 5001-801 Vila Real, Portugal; 3 Department of Pharmacy and Pharmaceutical Technology and Physical Chemistry, Faculty of Pharmacy, University of Barcelona, Ave. Joan XXIII s/n, 08028 Barcelona, Spain; 4 Department of Chemical and Instrumental Analysis, Faculty of Chemistry and Pharmacy, University of El Salvador, Final 25 Ave. Norte, 3026 San Salvador, El Salvador; 5 Centro de Investigaciones Químicas, Universidad Autónoma del Estado de Morelos, Av. Universidad No. 1001, Col Chamilpa, 62209 Cuernavaca, Mexico; 6 Department of Pharmaceutical Technology, Faculty of Pharmacy, University of Coimbra (FFUC), Pólo das Ciências da Saúde, 3000-548 Coimbra, Portugal; 7 CEB—Centre of Biological Engineering, University of Minho, Campus de Gualtar, 4710-057 Braga, Portugal

**Keywords:** oleanolic acid, ursolic acid, cytotoxicity, PLGA, polymeric nanoparticles, retinoblastoma cell line

## Abstract

Oleanolic (OA) and ursolic (UA) acids are recognized triterpenoids with anti-cancer properties, showing cell-specific activity that can be enhanced when loaded into polymeric nanoparticles. The cytotoxic activity of OA and UA was assessed by Alamar Blue assay in three different cell lines, i.e., HepG2 (Human hepatoma cell line), Caco-2 (Human epithelial colorectal adenocarcinoma cell line) and Y-79 (Human retinoblastoma cell line). The natural and synthetic mixtures of these compounds were tested as free and loaded in polymeric nanoparticles in a concentration range from 2 to 32 µmol/L. The highest tested concentrations of the free triterpene mixtures produced statistically significant cell viability reduction in HepG2 and Caco-2 cells, compared to the control (untreated cells). When loaded in the developed PLGA nanoparticles, no differences were recorded for the tested concentrations in the same cell lines. However, in the Y-79 cell line, a decrease on cell viability was observed when testing the lowest concentration of both free triterpene mixtures, and after their loading into PLGA nanoparticles.

## 1. Introduction

**Figure 1:**
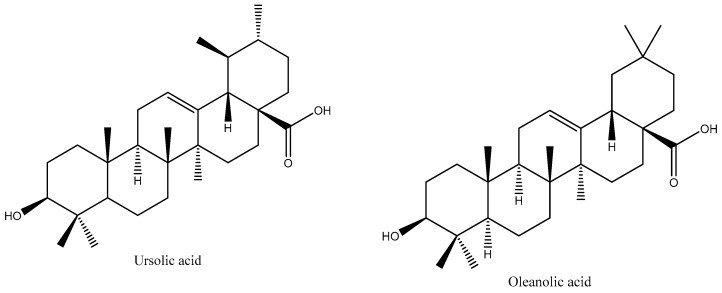
The chemical structure of ursolic acid (UA) and oleanolic acid (OA).

Pentacyclic triterpenes are known for their multiple pharmacological properties [1,2,3], which are attributed to their resemblance to biogenesis to steroids in the cyclization of squalene. As pentacyclic triterpenes, they exist in a range of medicinal plants, becoming an interesting source of new active ingredients for pharmaceutical applications [3]. In the present work, selected pentacyclic triterpenes have oleanane and ursane based structures, i.e., the oleanolic acid (OA) (of chemical name: 3β-3-hydroxyolean-12-en-28-oic acid) and its isomer, ursolic acid (UA) (of chemical name: 3β-3-hydroxyurs-12-en-28-oic acid). Both structures are shown in Figure 1. These compounds are found in a set of medicinal herbs and fruits [2,3,4,5]. While having interesting biological activities (anti-inflammatory, anticancer, hepatoprotective, antioxidant, antifungal, antimicrobial activity), their bioavailability is greatly hindered by their poor water solubility. Due to their similar chemical structure, the biological activity and therapeutic interest of OA and UA are rather similar, which is highly related to their dose-dependent cytotoxicity profile [6]. Previous studies have shown that these triterpenes exhibited cytotoxicity in several types of cancer cell lines [6,7,8]. Besides targeting tumor cells by induction of apoptosis, oleanolic and ursolic acids also modulate the tumor environment exhibiting antiangiogenic and anti-inflammatory activities, together with antioxidant effects and cell differentiation (e.g., [3]). As the efficacy of each single compound might differ with respect to the identified bioactivities, the combination of these substances in a single dosage form might have synergistic effects in the treatment of cancer [9].

Cytotoxicity assays are widely used by the pharmaceutical industry to screen the biosafety of compounds, and also to evaluate the potential anti-tumoral effect of chemotherapeutic drugs. Among the methodologies commonly used to evaluate cell viability, the authors can highlight the MTT (3-[4,5-dimethylthiazol-2-yl]-2,5-diphenyl tetrazolium bromide) [10,11], SRB (sulforhodamine B), [6,8], trypan blue [12], WST-8 (2-(2-methoxy-4-nitrophenyl)-3-(4-nitrophenyl)-5-(2,4-disulfophenyl)-2H-tetrazolium, monosodium salt) assay [13], clonogenic assay [11], Alamar blue [14,15,16,17,18], among others [19].

In the last decades, the success of chemotherapy has significantly increased by site specific targeting of anti-cancer drugs when loaded into nanoparticles [20]. These submicron-sized carriers offer advantages over standard dosage forms, with the possibility to overcome adverse side reactions and systemic toxicity. Tumor-targeted drug delivery increases intra-tumoral drug concentration while lowering the chemotherapeutic concentration in healthy tissues [21]. 

The loading of natural products in nanoparticles for anticancer therapy, as well as for the treatment of other diseases, is being widely explored with the aim to protect sensitive compounds from degradation, to increase their solubility and thus their bioavailability [22,23]. Nanoparticles have been used for targeting and delivery [24], to reduce inflammation or even facilitate the action of other drugs [25,26]. The evaluation of the cytotoxicity/safety of new drug-loaded nanoparticle formulations, and the comparison of drug effects after being encapsulated becomes therefore of crucial relevance. In this work, the cytotoxic profile of natural (NM) and synthetic (SM) mixtures of oleanolic acid (OA) and ursolic acid (UA) before and after their loading into poly (d,l-lactide-*co*-glycolide) acid (PLGA) nanoparticles (PLGA-NPs) have been reported. The Alamar blue assay was carried out using three different cell lines, i.e., human hepatoma (HepG2), human epithelial colorectal adenocarcinoma (Caco-2) and human retinoblastoma (Y-79). The characterization of the developed nanoparticle formulations was reported in a previous work [25], in which the stability of NPs over 6 months was studied at two temperatures (4 °C and 25 °C).Our results showed that, from month 0 to month 6: (i) Only a slight increase of Z-ave, from 220 nm to 230 nm (SM-NPs) and from 210 nm to 230 nm (NM-NPs), without changing polydispersity index (PI) at 4 °C (=0.08) for SM-NPs and with a slight increase, from 0.098 to 0.111, for NM-NPs, was observed; (ii) ZP decreased approximately 2–3 mV, (initial ZP was −27 mV and −25 mV, SM-NPs and NM-NPs, respectively); and (iii) release kinetics occurred over a period of 75 h for both formulations (i.e., 75% released). The reproducibility of NPs characteristics has also been reported [25,26,27]. Thus, this study aimed to compare the safety/cytotoxicity of OA/UA mixtures (of natural source obtained from *Plumeria obtusa* leaves extraction [26] and of synthetic source) with the corresponding encapsulated mixtures (SM-OA/UA NPs and NM-OA/UA NPs).

## 2. Materials and Methods 

### 2.1. Chemicals

Resomer^®^ RG756 S [poly (d,l-lactide-*co*-glycolide) acid (PLGA)] of 98,000 Da of molecular weight (MW) and 75/25 molar ratio of lactide/glycolide was a kind gift from Boehringer (Ingelheim, Germany). The selected triterpenoids, Oleanolic Acid (OA) (≥97%) and Ursolic Acid (UA) (≥90%) were purchased from Sigma-Aldrich (Madrid, Spain). Lutrol^®^ F68 (Poloxamer 188, P188) was offered by BASF (Barcelona, Spain). The reagents and consumables for the cell culture were purchased from Gibco (Alfagene, Invitrogen, Carcavelos, Portugal). MilliQ water was obtained by a MilliQ^®^ Plus System lab supplied. All other reagents were of analytical or high-performance liquid chromatography (HPLC) grade obtained from Fisher Scientific (Leicestershire, UK).

### 2.2. Production of Natural (NM) and Synthetic (SM) Mixtures of Pentacyclic Triterpenes

The leaves of *Plumeria obtusa* L. var. *sericifolia* (C. Wright ex Griseb.) Woodson (Apocynaceae), collected in Calakmul, Campeche, Mexico [28] were used as a source of natural mixture of oleanolic and ursolic acids (NM-OA/UA). Briefly, the methanol extract of leaves was processed in a column chromatography under reduced pressure, and eluted with mixtures of hexane/ethyl acetate of increasing polarity. A 50:50 hexane/ethyl acetate chromatography elution system was used to obtain a POLHC-1OA fraction, as a white amorphous powder of 250–260 °C of melting point. The retention factor (Rf) was 0.63. The natural mixture (NM-OA/UA) was obtained with a purity of 58% and analyzed by HPLC to determine the ratio between each triterpene. A synthetic mixture (SM-OA/UA), using the two triterpenes acquired from Sigma-Aldrich, was further prepared, in the same proportion of oleanolic and ursolic acids as found in NM-OA/UA (1:9 *w*/*w*).

### 2.3. Preparation of Nanoparticles

PLGA-NPs loaded with NM-OA/UA or SM-OA/UA were prepared applying the solvent displacement production procedure [29]. Briefly, 90 mg PLGA was dissolved in 5 mL of acetone containing 1.0 mg/mL of mixture (NM or SM). This organic solution was poured into 10 mL of an aqueous solution, adjusted to pH 5.5, of 10 mg/mL poloxamer 188 (P 188), under moderate stirring. Acetone was then evaporated and PLGA-NPs dispersions were then concentrated under reduced pressure, as described [26].

### 2.4. Cell Culture

Y-79 (Human retinoblastoma cell line) and Caco-2 (Human epithelial colorectal adenocarcinoma cell line) were purchased from Cell Lines Service CLS (Eppelheim, Germany). HepG2 (Human hepatoma cell line) was purchased from ATCC (LGC Standards S.L.U., Andorra, Spain). The adherent cell lines (Caco-2 and HepG2) were maintained in DMEM (Dulbecco’s Modified Eagle Medium; Gibco, Alfagene, Portugal), whereas Y-79 cell line was maintained in RPMI-1640 (Roswell Park Memorial Institute medium). Both media were supplemented with 10% (*v*/*v*) FBS (fetal bovine serum), 2 mM l-glutamine, and antibiotics (100 U/mL penicillin and 100 µg/mL of streptomycin), in an incubator with atmosphere of 5% CO_2_ in air at 37 °C, as previously described [14,17].

### 2.5. Cytotoxicity Assay

For the cytotoxicity assays, the adherent cells (Caco-2 and HepG2) were handled as described in [17] and the non-adherent Y-79 cells, that grow in suspension forming aggregates, were handled as described in [14] and then seeded into previously poly-l-Lysine coated 96-well plates. Briefly, the three cell lines were counted and, after appropriate dilution in culture media, were seeded at 5 × 10^4^ cells/mL (Caco-2 and HepG2) or 1 × 10^5^ cells/mL (Y-79) density, in 96-well plates (100 µL/well). The stock solutions of NM-OA/UA and SM-OA/UA were prepared in dimethyl sulfoxide (DMSO) at a concentration of 1.0 µg/µL (2.2 mM). For each mixture and formulation (NM-OA/UA, SM-OA/UA, NM-OA/UA NPs, SM-OA/UA NPs and blank-NPs), five concentrations (2, 4, 8, 16 and 32 µmol/L) were tested, by diluting the required volume of NM or SM with FBS-free culture media to achieve the final concentrations, or the appropriate volume of NPs that contain the required volume of OA/UA, and added to cells 24 h after seeding (100 µL/well). The cell viability was evaluated, after 24 h or 48 h of cells exposure to above mentioned solutions, by applying the Alamar blue assay (Alfagene, Invitrogen, Carcavelos, Portugal). The sample solutions were replaced by a 10% (*v*/*v*) Alamar blue solution, diluted in FBS-free culture medium, adding 100 µL to each well. Absorbance was read at 570 nm (reduced form; resorufin) and 620 nm (oxidized form; resazurin). The data were analyzed by calculating the percentage of Alamar blue reduction (according to the manufactures recommendation) and expressed as a percentage of the control (untreated cells), as previously reported [30].

### 2.6. Statistical Analysis

The data presented are the mean ± standard deviation of four determinations, following analysis of variance (ANOVA) in the GraphPad Prism software (version 5.01). The Tukey multiple comparison test was used to compare the significance of the difference between the groups, a *p*-value < 0.05 was accepted as significant.

## 3. Results and Discussion

### 3.1. Characterization of Nanoparticles

Submicron-sized PLGA-NPs were produced by a solvent displacement procedure. A mean particle size of 213.55 ± 1.60 nm was obtained for the NM-OA/UA NPs, with a polydispersity index of 0.090 ± 0.038 and zeta potential of −27.12 ± 0.27 mV. For SM-OA/UA NPs, a mean particle size of 217.98 ± 2.74 nm, polydispersity index of 0.073 ± 0.011 and zeta potential of −26.85 ± 0.49 mV were recorded. The encapsulation efficiency reached 79.01 ± 4.22% for NM-OA/UA NPs and 78.25 ± 3.01% for SM-OA/UA NPs. Both formulations exhibited a Newtonian behavior and high long-term physical stability, without presenting phenomena of destabilization over a period of 6 months. The production of these NPs has been optimized using a 2^3^ factorial design, as reported in Alvarado et al. [25], and the used methodology provides particles with reproducible characteristics. The NPs presented a spherical-shape with identical sizes (~200 nm) for both mixtures, as reported [25]. In a previous in vitro study, these NPs showed to have identical release kinetics, being the release from NM-OA/UA NPs slightly higher up to 40 h of assay, and attaining nearly maximal and identical % release values from 40 h to 72 h (about 75% [25]). This formulation exhibited enhanced OA/UA corneal permeation, showing an identical cumulative permeated amount for both mixtures over time [25]. With this in mind, aiming to use these drugs for ocular application or oral administration, the cytotoxicity of blank NPs, individual mixtures and the loaded NPs were evaluated.

### 3.2. Cytotoxicity Assay

Alamar blue was selected for the evaluation of the pentacyclic triterpenoids cytotoxicity, as it is a quantitative measure of cell proliferation and metabolic activity, useful in the cytotoxicity screening of tested compounds/formulations and can be used as a baseline for further in vivo studies. Alamar blue is a reliable, simple, non-radioactive, sensitive fluorometric/colorimetric assay commonly used to detect the metabolic activity of cells. This assay combines additional advantages, as it is ready to use, extremely stable, minimally toxic to the cells, and allows for the continuous monitoring of cultures. It may also be useful to predict if formulations cause cellular damage which can result in the loss of the metabolic cell function. Indeed, the oxidation-reduction (REDOX) indicator is incorporated in the cell which, when under a reducing environment of a metabolically active cell, is reduced. Alamar blue becomes therefore fluorescent and changes its color from blue to pink. The reduction of Alamar blue can be mediated by mitochondrial enzymes or cytosolic and microsomal enzymes [14,31,32]. The cytotoxic activity of free and encapsulated mixtures of OA/UA was evaluated on three human cell lines, HepG2, Caco-2 and Y-79. 

**Figure 2:**
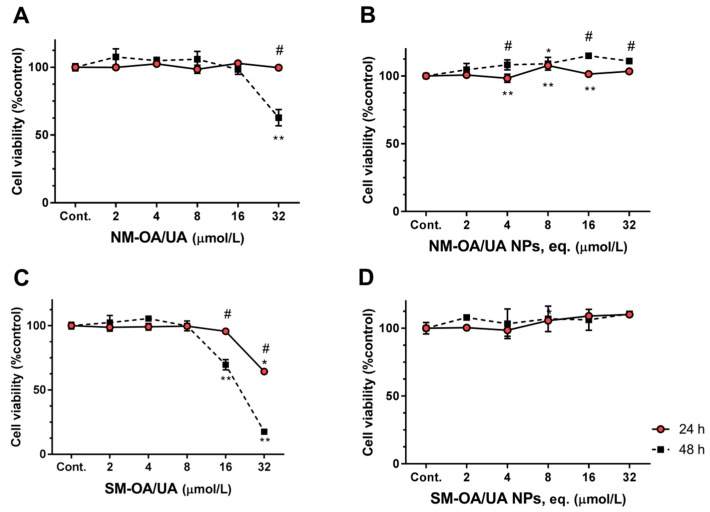
The effect of OA/UA on HepG2 (human hepatoma) cells viability. HepG2 cells were exposed to the natural mixture of oleanolic and ursolic acids (NM-OA/UA) (**A**), NM-OA/UA nanoparticles (NPs) (**B**), the synthetic mixture of oleanolic acid and ursolic acids (SM-OA/UA) (**C**), SM-OA/UA NPs (**D**), for 24 and 48 h, as denoted. Statistically significant differences, *p* < 0.05, are denoted with * or **, in relation to respective control at 24 or 48 h, respectively; # denotes statistical differences for incubation periods at indicated concentration. The results are the mean ± S.D. of *n* = 4.

Figure 2 shows the cell viability in HepG2 cell line after 24 and 48 h of exposure to different concentrations of both mixtures of OA/UA and of the mixtures loaded into PLGA nanoparticles. After 24 h of exposure, at concentrations up to 16 µmol/L, the pure mixture of triterpenoid acids (NM-OA/UA; Figure 2A) did not produce significant changes on the cell viability when compared to the control (untreated cells). SM-OA/UA (Figure 2B) at the highest concentration (32 µmol/L) produced a decrease of approximately 35% on the cell viability (cell viability is 65.23% of control). The nanoparticles loading the mixtures showed no changes (Figure 2B,D). After 48 h of exposure, both pure mixtures (i.e., non-loaded mixtures) showed a fairly significant decrease on the cell viability, i.e., at 32 µmol/L cell viability reached values of 62.89% for NM-OA/UA (Figure 2A) and 17.52% for SM-OA/UA (Figure 2C), compared to the unexposed control. However, no significant negative changes on the cell viability were recorded when loading the mixtures in the NPs, demonstrating that NPs reduce drug toxicity at the highest tested concentration.

**Figure 3:**
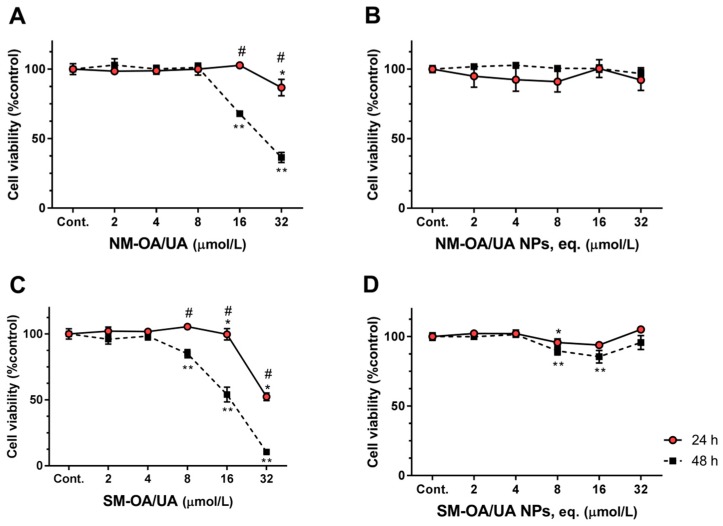
The effect of OA/UA on Caco-2 (human epithelial colorectal adenocarcinoma) cells viability. Caco-2 cells were exposed to NM-OA/UA (**A**), NM-OA/UA NPs (**B**), SM-OA/UA (**C**), SM-OA/UA NPs (**D**), for 24 and 48 h, as denoted. Statistically significant differences, *p* < 0.05 are denoted with * or **, in relation to respective control at 24 or 48 h, respectively; # denotes statistical differences for incubation periods at indicated concentration. The results are the mean ± S.D. of *n* = 4.

The cell viability in Caco-2 cells is shown in Figure 3, where similar results to the HepG2 cells are observed. After 24 h of exposure, the pure mixtures of both natural and synthetic triterpenes showed no significant changes from the control in concentrations from 2 to 16 µmol/L (NM-OA/UA) and from 2 to 8 µmol/L (SM-OA/UA). However, a reduction on cell viability to the concentration of 32 µmol/L was observed, where a decrease of approximately 14% and 48% (cell viability of 86.8% and 52.30%) for NM-OA/UA (Figure 3A) and SM-OA/UA (Figure 3C) respectively, was observed. The mixtures encapsulated in PLGA-NPs produced no significant changes on the cell viability at all concentrations (Figure 3B,D). Concerning the 48 h exposure (Figure 3, square symbols), Figure 3C shows that SM-OA/UA produced a significant reduction on the cell viability, starting from the concentration of 8 µmol/L and reaching a value of 10.41% (~90% reduction) at the concentration of 32 µmol/L. NM-OA/UA (Figure 3A) only started producing a significant reduction on the cell viability at 16 µmol/L, reaching 36.5% at 32 µmol/L (reduction of 63.5% on the cell viability in comparison to the control). As in the case of the HepG2 cells, OA/UA mixtures loaded into NPs produced no significant changes on the cell viability compared to control cells, showing again that NPs decreased the toxicity of the compounds.

The obtained data demonstrate that these terpenoids have cytotoxic activity against HepG2 and Caco-2 cells, which may translate to anti-tumoral activity. The toxicity of these compounds was significantly reduced or abolished when loaded into PLGA nanoparticles. This was attributed to a controlled release leading to concentrations onto the cells, that were not enough to exhibit cytotoxic effects but that could have a protective role. These results may demonstrate that the developed formulation exhibit some protective effects in the intestine and liver as HepG2 and Caco-2 cells are in vitro models for these organs, respectively. This might be a promising result for the formulation of these drugs in innovative oral dosage forms.

Y-79 is a human retinoblastoma (RB) cell line that has been firstly established from an explant of a primary tumor of a donor with a family history of retinoblastoma. The cultured tumoral cells had ultrastructural characteristics similar to those seen in the original tumor, such as microtubules, triple membrane structures, nuclear membrane infoldings, centrioles, large coated vesicles, basal bodies and annulate lamellae [33]. The incidence of retinoblastoma remains constant worldwide at one case per 15,000–20,000 live births, which corresponds to approximately 9000 new cases every year [34,35]. Retinoblastoma is the most common malignant eye tumor diagnosed in childhood and can occur as heritable (usually bilateral) and non-heritable (unilateral) forms in infants [36]. In health conditions, retinoblasts suffer division into new cells and fill the retina, dividing and developing into mature retinal cells. This occurs during early stages of human development in the womb. Under pathological conditions, instead of maturing into special cells that detect light, some retinoblasts continue to divide and grow out of control, forming the retinoblastoma. The retinoblastoma gene (*RBl*) is a molecular marker of retinoblastoma tumors. This gene is located in chromosome 13q14. 

**Figure 4:**
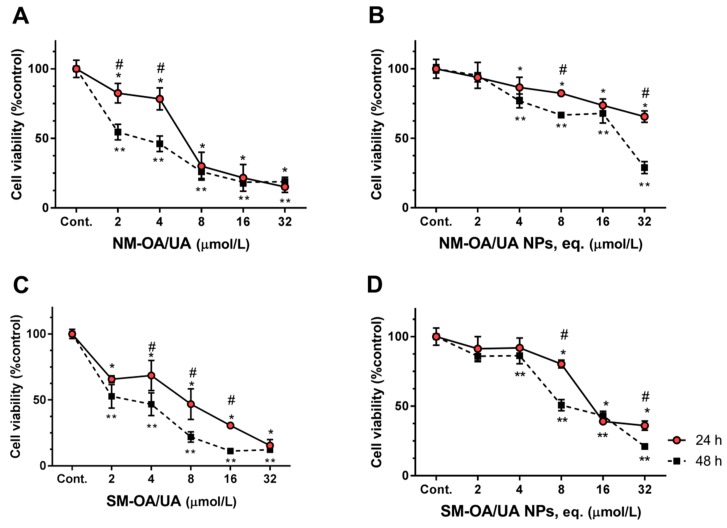
The effect of OA/UA on Y-79, human retinoblastoma cells viability. Y-79 cells were exposed to NM-OA/UA (**A**), NM-OA/UA NPs (**B**), SM-OA/UA (**C**), SM-OA/UA NPs (**D**), for 24 and 48 h, as denoted. Statistically significant differences, *p* < 0.05 are denoted with * or **, in relation to respective control at 24 or 48 h, respectively; # denotes statistical differences for incubation periods at indicated concentration. The results are the mean ± S.D. of *n* = 4.

Figure 4 shows the results of the cell viability using Y-79 cell line. Further, 24 h after applying the formulations to the cells, non-loaded mixtures produced significant reductions on the cell viability. From the first concentration of 2 µmol/L to the highest tested concentrations (32 µmol/L), the cell viability values reached 15.16% and 15.43% for NM-OA/UA (Figure 4A) and SM-OA/UA (Figure 4C), respectively, (a reduction on cell viability of approximately 85%). While NPs decreased the toxicity of triterpenoid compounds in HepG2 and Caco-2 cell lines, in the Y-79, the loaded mixtures still produced a significant reduction on the cell viability. From 4 µmol/L to the highest tested concentration (32 µmol/L), the values of the cell viability reached ca. ~65.62% for NM-OA/UA NPs (Figure 4B) and 36.06% for SM-OA/UA NPs (Figure 4D). After 48 h of exposure, a significant decrease on the cell viability, from the lowest concentration, reached 32 µmol/L values of the cell viability of 18.84% for NM-OA/UA, 12.24% for SM-OA/UA, 28.97% for NM-OA/UA NPs and 21.01% for SM-OA/UA NPs.

**Figure 5:**
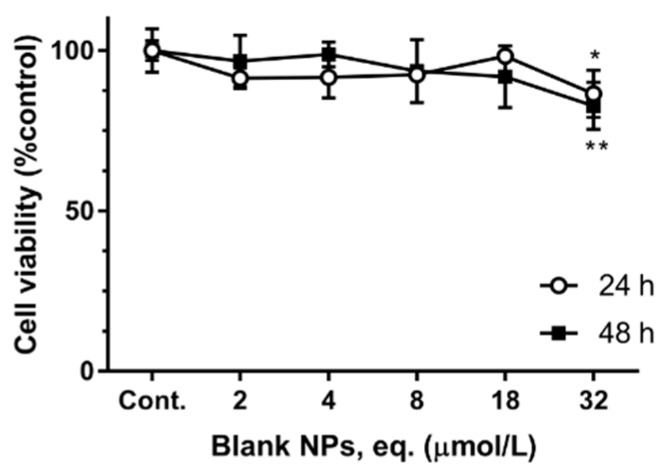
The effect of blank NPs on Y-79 cell viability. Y-79 cells were exposed to blank NPs, for 24 and 48 h, as denoted. Statistically significant differences, *p* < 0.05, are denoted with * or **, in relation to respective control at 24 or 48 h, respectively. The results are the mean ± S.D. of *n* = 4.

The effect of blank-NPs on the cell viability of all the cell lines was tested at the two time-points and showed to be statistically different from the control. However, the observed decrease was only approximately 15% of the control (cell viability ~85–90%, at highest concentrations). Figure 5 shows the results obtained for Y-79 cells exposure to blank-NPs as these cells showed the most notorious effect of the encapsulated triterpene mixtures, showing a cell viability of 82 ± 7% of the control, after 48 h exposure to 32 µmol/L (equivalent of blank NPs). At 48 h exposure to 32 µmol/L equivalent of blank NPs, the viability of Caco-2 and HepG2 cells was 88% and 95%, respectively (data not shown). These small reductions on the cell viability is considered non-toxic [19], making these NPs safe for drug delivery.

These decreases on the cell viability, especially pronounced in Y-79 cell line, are possible because these compounds exhibit potential anticancer effects both in vitro and in vivo. They inhibited proliferation and caused apoptosis in the cells of numerous cancers, including melanoma, pancreatic cancer, breast cancer, lung cancer, colon cancer, cervical cancer [37,38,39,40,41]. However, most of their mechanisms of action are still unclear. Further, UA and OA have been described as having cytostatic activity by inducing cell cycle arrest in G0/G1 phase [42], by inducing apoptosis through generating reactive oxygen species (ROS) and caspase signaling pathways [3]. *Thymus* plant extracts, rich in UA and OA triterpenoids (~12% *w*/*w*; with UA:OA of ~2:1) were also reported to exhibit high anti-proliferative effects through cell cycle arrest and apoptosis [18]. From these data, it can be seen that different cell lines exhibit different sensitivity both to the unloaded and to the loaded OA/UA mixtures (see Table 1). The OA/UA mixtures produced a more pronounced cytotoxic effect than the corresponding NPs. This might result from the release kinetics of these systems. Indeed, Alvarado et al. [25] reported that NM-AO/UA NPs and SM-AO/UA NPs showed approximately 50% and 75% release, respectively, at 24 h, and both presented 75% release at 48 h. As the UA and OA content in NM-AO/UA and SM-AO/UA is immediately accessible to cells, it is expected that their action is more pronounced, as it is demonstrated by the significantly lower IC_50_ (Table 1), compared to the encapsulated ones. The synthetic mixture (SM-AO/UA) seems to be slightly more effective in reducing the cell viability than the natural one. This may result from the higher purity of purchased compounds (named as synthetic; see material) in comparison to the ones obtained by extraction, as referred in Alvarado et al. [26]. The lower purity of NM-OA/UA, in comparison to SM-OA/UA, might also explain the reason why SM-OA/UA produced lower IC_50_, for HepG2 and Caco-2 than NM-OA/UA (Table 1). Concerning Y-79 cells, it was observed that they are very sensitive to these triterpenoids as the IC_50_ of both mixtures are very low (Table 1), indicating a high toxicity as shown in Figure 4. In these cells, as they are very sensitive to these triterpenoids, the encapsulated triterpenoids exhibit an anti-proliferative effect over time. Most natural bioactive compounds have a dualistic effect, depending on the concentration and mode of action. In this case, by controlling drug release, NPs promote the protection of Caco-2 (colon cells) and HepG2 (hepatic cells) which are not that sensitive to these compounds while promoting efficient cell viability reduction in Y-79 cells. They are also capable of exhibiting anticancer effects as these are more sensitive to OA/UA triterpenoids. Cell specific-sensitivity to drugs and natural compounds has been widely reported. From the experience of the authors, *Thymus* plant extracts, rich in UA and OA, induce very low toxicity in Caco-2 and HepG2 cells [18] compared to MCF-7 (breast cancer cells), BT-474 (breast cancer cells) and Raw 264.7 cells [4], being the Raw 264.7 cells IC_50_, for 24 h exposure, of 24 µg/mL of extract (~12% of the extract is UA and OA) [4].

## 4. Conclusions

In summary, oleanolic (OA) and ursolic (UA) acids have some toxicity at high concentrations (anticancer effect), especially pure (non-loaded) synthetic mixtures which show higher toxicity than the natural mixture in all cell lines. This is attributed to the purity or to the presence of other isomer with lower activity than UA or OA. The potent cytotoxic activity against Y-79 cell line exhibited by these compounds is a promising result to develop new formulations for the treatment of retinoblastoma as prospective anticancer agents, as previous studies also showed them to have a good anti-inflammatory and antioxidant activity. The lower toxicity against Caco-2 cells (colon) and HepG2 (liver) is also very important as it opens new perspectives in oral-delivery formulations with low toxicity, and also ensures low enteric toxicity if inadvertently ingested. 

## Figures and Tables

**Table 1:** The values of IC_50_ (µmol/L), (half maximal inhibitory concentration) obtained for HepG2, Caco-2 and Y-79 cells exposed NM-OA/UA, SM-OA/UA, NM-OA/UA NPs and SM-OA/UA NPs. The results are expressed as the mean ± SD, *n* = 4.

		IC_50_ (µmol/L)			
		NM-AO/UA	SM-AO/UA	NM-AO/UA NPs	SM-AO/UA NPs
**HepG2**	24 h	>32.0	>32.0	>>32.0	>>32.0
	48 h	>32.0	24.3 ± 1.8	>>32.0	>>32.0
**Caco-2**	24 h	>32.0	32.4 ± 0.9	>32.0	>32.0
	48 h	24.3 ± 0.9	16.3 ± 0.6	>32.0	>32.0
**Y-79**	24 h	6.4 ± 0.9	6.5 ± 0.9	82.0 ± 10.2	16.6 ± 1.3
	48 h	2.6 ± 0.4	2.9 ± 0.3	19.1 ± 2.2	11.1 ± 0.8

Abbreviation: IC_50_, half maximal inhibitory concentration. In table, > denotes higher than; >> denotes much higher than.
